# Indoxyl sulfate promotes apoptosis in cultured osteoblast cells

**DOI:** 10.1186/2050-6511-14-60

**Published:** 2013-12-01

**Authors:** Young-Hee Kim, Kyung-Ah Kwak, Hyo-Wook Gil, Ho-Yeon Song, Sae-Yong Hong

**Affiliations:** 1 Department of Microbiology, Soonchunhyang University Medical college, Cheonan, Korea; 2 Department of Internal Medicine, Soonchunhyang University Cheonan Hospital, 31 Soonchunhyang 6gil, Dongnam-gu, Cheonan, Chungnam 330-721, Korea

**Keywords:** Uremia, Renal osteodystrophy, Apoptosis, Cell differentiation, Organic anion transporters

## Abstract

**Background:**

Indoxyl sulfate (IS), an organic anion uremic toxin, promotes the progression of renal dysfunction. Some studies have suggested that IS inhibits osteoclast differentiation and suppresses parathyroid hormone (PTH)-stimulated intracellular cAMP production, decreases PTH receptor expression, and induces oxidative stress in primary mouse calvaria osteoblast cell culture. However, the direct effects of IS on osteoblast apoptosis have not been fully evaluated. Hence, we investigated whether IS acts as a bone toxin by studying whether IS induces apoptosis and inhibits differentiation in the cultured osteoblast cell line MC3T3-E1.

**Methods:**

We assessed the direct effect of IS on osteoblast differentiation and apoptosis in the MC3T3-E1 cell line. We examined caspase-3/7 activity, apoptosis-related proteins, free radical production, alkaline phosphatase activity, and mRNA expression of type 1 collagen and osteonectin. Furthermore, we investigated the uptake of IS via organic anion transport (OAT).

**Results:**

We found that IS increased caspase activity and induced apoptosis. Production of free radicals increased depending on the concentration of IS. Furthermore, IS inhibited the expression of mRNA type 1 collagen and osteonectin and alkaline phosphatase activity. The expression of OAT, which is known to mediate the cellular uptake of IS, was detected in in the MC3T3-E1 cell line. The inhibition of OAT improved cell viability and suppressed the production of reactive oxygen species. These results suggest that IS is transported in MC3T3-E1 cells via OAT, which causes oxidative stress to inhibit osteoblast differentiation.

**Conclusions:**

IS acts as a bone toxin by inhibiting osteoblast differentiation and inducing apoptosis.

## Background

Indoxyl sulfate (IS) is an organic anion uremic toxin belonging to the family of protein-bound retention solutes [1]. IS is synthesized in the liver from indole, which is produced from the metabolism of dietary tryptophan in the body. The studies performed to date have shown that IS accumulates in blood and promotes the progression of renal dysfunction [2-5]. IS may also act as a vascular toxin [2-4,6]. It directly stimulates rat vascular smooth muscle cell proliferation in a concentration-dependent manner. Furthermore, Dahl salt-sensitive hypertensive rats administered IS in combination with a high-salt diet have been found to show an increase in aortic wall thickness and severe aortic calcification, with colocalization of osteoblast-specific proteins such as Cbfa-1, osteonectin, and alkaline phosphatase [7]. In a recent study, Iwasaki et al. reported that when rats with renal dysfunction and low bone turnover were administered an oral adsorbent, their blood IS level decreased and osteoblastic cell function improved [8]. Iwasaki et al. have also shown that in primary mouse calvaria osteoblast cell culture, addition of IS suppresses parathyroid hormone (PTH)-stimulated intracellular cAMP production, decreases PTH receptor expression, and induces oxidative stress [9]. IS inhibits osteoclast differentiation and bone-resorbing activity, which could affect bone remodeling in chronic kidney disease patients [10]. Limited data suggest that IS could act as a bone toxin by affecting both osteoblast and osteoclast activities. To date, the direct effects of IS on osteoblast apoptosis have not been fully evaluated.

Hence, we investigated whether IS acts as a bone toxin by studying whether IS induces apoptosis and inhibits differentiation in a cultured osteoblast cell line.

## Methods

### Chemicals

l-Ascorbic acid, β-glycerophosphate, probenecid, probucol, *N*-acetylcysteine (NAC), and IS were all obtained from Sigma (St. Louis, MO, USA). All cell culture media and supplements were from Hyclone (Logan, UT, USA). Reagents for reverse transcription and those for real-time PCR reactions were from Toyobo (Osaka, Japan). Anti-Bax, Anti-Bcl-2, and anti-p53 mouse monoclonal antibodies were purchased from Santa Cruz (Santa Cruz, CA, USA). Secondary goat anti-rabbit IgG was obtained from Thermo Fisher Scientific (Rockford, USA). The assay kit for caspase-3/7 activity was purchased from Promega (Mannheim, Germany).

### Cells and osteogenic induction

Newborn mouse calvaria-derived MC3T3-E1 subclone 14 pre-osteoblastic cells (ATCC, USA) were cultured in α-MEM medium (Hyclone) supplemented with 10% fetal bovine serum (Hyclone), 100 U/mL penicillin, and 100 mg/mL streptomycin (Hyclone) at 37°C in an atmosphere with 100% humidity and 5% CO_2_. Osteoblast differentiation was induced by the addition of 10 mM β-glycerophosphate, as described previously [11].

### Cell viability

Cell viability was assessed using the 3-(4,5-dimethylthiazol-2-yl)-2,5-diphenyltetrazolium bromide (MTT) assay, as described previously [12]. MC3T3-E1 cells were incubated in osteogenic induction medium with or without IS at 37°C for 72 h. After the cells were lysed with DMSO solution, the optical density was measured at 590 nm using the optical density at 630 nm as reference (VICTOR™X3; PerkinElmer, USA).

### Bone differentiation

Alkaline phosphatase (ALP) activity was measured in cells treated with 0–1.5 mM IS and in control cells incubated for 3, 5, 7, and 10 d. Cells were washed with PBS and were lysed with a solution containing 0.1% Triton X-100 at the same time as the cellular alkaline phosphatase activity and cell protein content were determined. The enzymatic reaction was started by the addition of 50 μL of substrate/buffer mixture (equal volumes of *p*-nitrophenol phosphate substrate [N 1891; Sigma Chemicals, St. Louis, MO] and alkaline buffer solution [A9226; Sigma Chemicals]). After 30 min of incubation at 37°C, the reaction was stopped by adding an equal volume of 0.05 M NaOH. The lysate from the wells was collected into individual Eppendorf tubes and vortexed. The ALP activity was determined colorimetrically at 405 nm using *p*-nitrophenol (PNP) standards (0–50 nmol, N7660; Sigma Chemicals). The protein concentration in the lysate was determined using the Bradford assay. ALP activity is expressed as nanomoles of PNP released per milligram of protein.

### Assessment of cellular oxidative stress

Production of intracellular reactive oxygen species was detected using the nonfluorescent cell-permeating compound, 2′-7′-dichlorofluorescein diacetate (DCF-DA). DCF-DA is hydrolyzed by intracellular esterases, and is then oxidized by reactive oxygen species (ROS) to a fluorescent compound, 2′-7′-dichlorofluorescein (DCF). After treatment with 0–1.5 mM IS, MC3T3-E1 cells were treated with DCF-DA (10 μM) for 30 min at 37°C. Following DCF-DA exposure, the cells were rinsed and then scraped into PBS with 0.2% Triton X-100. Fluorescence was measured with a plate reader (VICTOR™X3) with excitation at 485 nm and emission at 535 nm.

### Flow cytometry analysis of apoptosis

Quantification of cells undergoing programmed cell death was conducted using an annexin V-propidium iodide apoptosis kit (Invitrogen). Analyzed cells were washed once in phosphate-buffered saline and resuspended in the binding buffer provided. Annexin V (Alexa 488-conjugated) and propidium iodide were added and incubated for 15 min at room temperature in the dark. The cells were analyzed using a FACS Calibur flow cytometer and CellQuest software.

### Apoptosis measurement: caspase-3/7 activity, and immunoblot assay for apoptosis-related factors p53, Bcl-2, and Bax

Caspase-3/7 activity was detected using a Caspase-Glo 3/7 Assay system (Promega) after preincubating the MC3T3-E1 cells (2 × 10^5^/96-well plate), followed by treatment with various IS concentrations (control, 0.5 mM, 1 mM) for 3, 6, 9, 12, and 24 h. The background luminescence associated with the cell culture and assay reagent (blank reaction) was subtracted from the experimental values. The activity of caspase-3/7 is presented as the mean value of triplets for the given cells. The intensity of the emitted fluorescence was determined at a wavelength of 521 nm with the use of luminometry (VICTOR™X3).

The immunoblot assay was conducted as follows. After stimulation, cells were washed once with phosphate-buffered saline and lysed with radioimmunoprecipitation assay (RIPA) lysis buffer (ROCKLAND, USA) and placed on ice for 30 min. Total cell extracts were centrifuged at 14 000 *g* (for 20 min at 4°C), and protein-containing supernatants were collected. Equal amounts of proteins (40 μg) were resolved by sodium dodecyl sulfate–polyacrylamide gel electrophoresis, transferred to a nitrocellulose membrane, and immunoblotted with specific antibodies against Bax, Bcl-2, and p53. Secondary antibodies were obtained from Thermo Fisher Scientific. Equal loading was confirmed using a β-actin antibody. Protein expression levels were quantified using a densitometer (ChemiDoc™ XPS + with Image Lab™ Software, Bio-Rad). The data are represented as the ratio of expression of the target protein to that of β-actin.

### RNA isolation, cDNA synthesis, and PCR analysis

Total RNA was isolated using an RNeasy Mini Kit (QIAGEN, Tokyo, Japan) according to the manufacturer’s instructions. Total RNA (1 μg) was used as the template for cDNA synthesis in a 50-μL reaction mixture using a reverse transcriptase-PCR kit (TOYOBO) according to the manufacturer’s instructions. Real-time PCR was performed on a CFX96™ (BIO-RAD). The PCR reactions consisted of Power SYBR Green PCR Master Mix (Applied Biosystems, UK), 0.1 mM (10 pM) specific primers, and 50 ng of cDNA. The primer sequences, designed with Beacon Designer 7.6 software (Bio-Rad), were as follows: mouse osteonectin, 5′-TCTCAACAAACAAATCAGGGAT-3′ and 5′-TGGCAGCACATTCATCTATG-3′; collagen 1, 5′-ATCACCAAACTCAGAAGATGTAG-3′ and 5′-CAGGAAGTCCAGGCTGTC-3′; organic anion transport 1 (OAT1), 5-ATG CCT ATC CAC ACC CGT GC-3 and 5-GGC AAA GCT AGT GGC AAA CC-3); OAT3, 5-CAG TCT TCA TGG CAG GTA TAC TGG-3 and 5-CTG TAG CCA GCG CCA CTG AG-3; and GAPDH, 5′-CAAGAAGGTGGTGAAGCA-3′ and 5′-TGTTGAAGTCGCAGGAGA-3′.

### Statistical analysis

All results are expressed as the mean ± standard error of the mean (SEM) values. The mean values of the groups were compared by analysis of variance, and a *P*-value <0.05 was considered significant.

## Results

### Effect of indoxyl sulfate on cell viability in the MC3T3-E1 cell line

**Figure 1:**
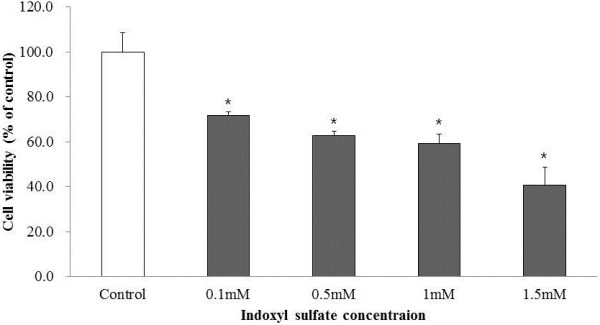
**Effect of IS on the viability of MC3T3-E1 cells.** Cell number was measured 24 h after the addition of IS at concentrations ranging from 0.1 to 1.5 mM and was expressed as the percentage of control cells not pretreated with IS (open bar). The cell toxicity of IS was found to be dose dependent. The data represent the mean ± SEM from 8 replicates in each group. *P < 0.05 vs. control cells.

To determine cytotoxicity, the effect of IS on the cell proliferation of MC3E3-T1 was studied using an MTT assay. As shown in Figure 1, IS, at the concentration range of 0.1–1.5 mM, inhibited cell proliferation at 72 h.

### Gene expression of OATs in the MC3T3-E1 cell line

**Figure 2:**
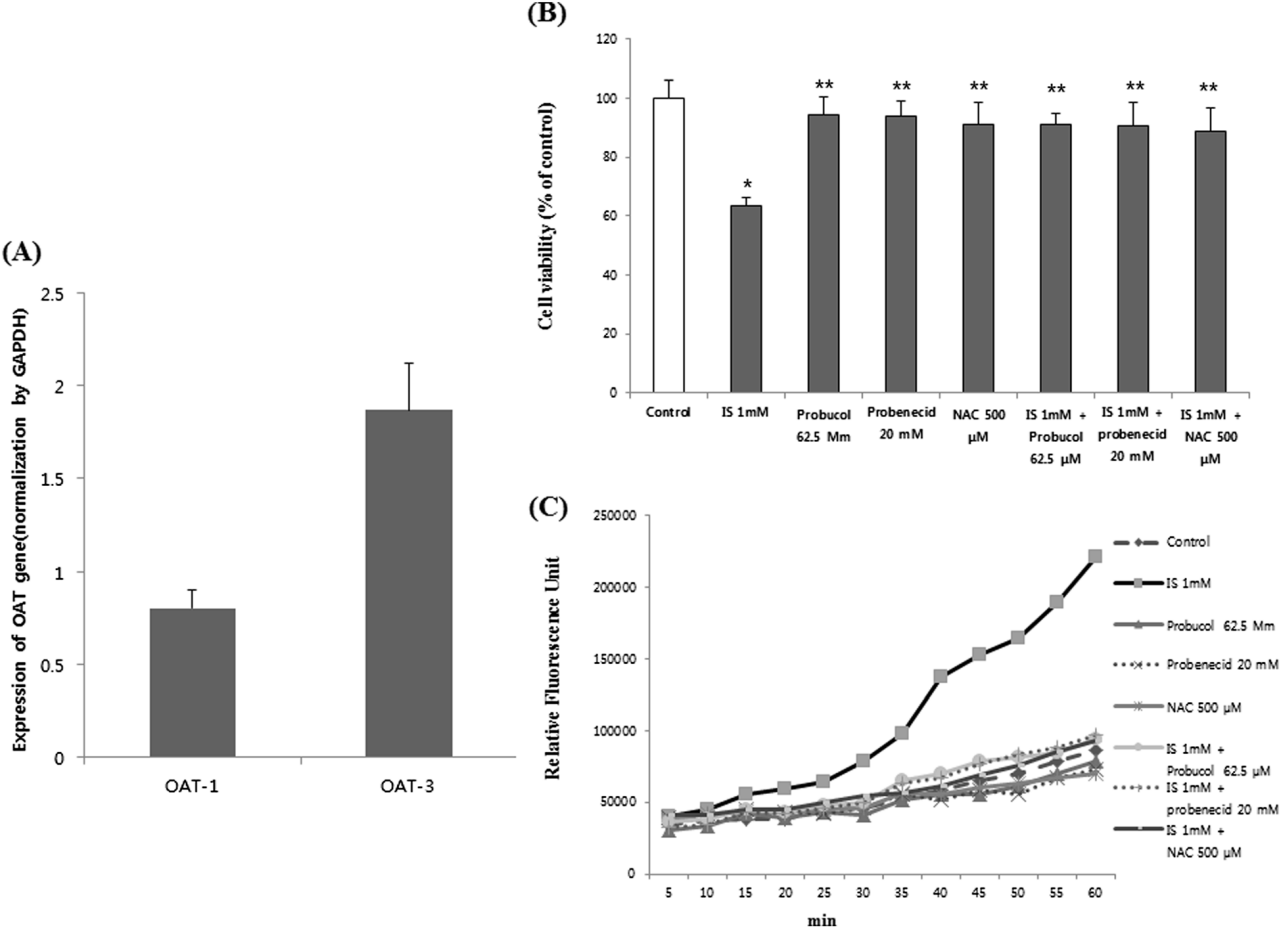
**Expression of the OAT gene in MC3T3-E1. (A)** OAT gene expression was determined by real time-polymerase chain reaction using RNA extracts. The expression of OAT-3 was relatively higher than that of OAT-1. The data represent the mean ± SEM (n = 3 for each group). **(B)** Probenecid (20 mM), an OAT inhibitor, reduced IS-induced cell toxicity, which is similar to an antioxidant effect. OAT is an IS transporter in MC3T3-E1 cells. The data represent the mean ± SEM from six determinations in each group. **(C)** IS-induced free radical production was suppressed by probenecid and antioxidants. The data represent the mean from six replicates in each group. OAT, organic anion transport; IS: indoxyl sulfate. *P < 0.05 vs control, ** *P* < 0.05 vs IS.

Because other studies have shown that IS is transported into osteoblasts and renal tubule cells via OAT, gene expression of OAT-1 and OAT-3 was investigated by real time PCR using RNA extracts. The expression of OAT-3 was relatively higher than that of OAT-1 in the MC3T3-E1 cell line (Figure 2A). After confirming the expression of the OAT gene in the MC3T3-E1 cell line, we investigated whether blocking OAT could prevent IS toxicity. To confirm the role of OAT in MC3T3-E1 cells, probenecid, a transporter inhibitor, was added to the cells during pretreatment with 1 mmol/L of IS. Blocking OAT in MC3T3-E1 cells improved cell survival (Figure 2B). ROS production was inhibited by probenecid. The effect obtained was similar to that obtained on pretreatment with the antioxidants NAC (500 μM) and probucol (62.5 μM) (Figure 2C)*.* Probenecid works by interfering with the OAT in the kidneys, which blocks the efflux of IS in cells. Probucol is a phenolic lipid-lowering agent with antioxidant and anti-inflammatory properties. *N*-acetylcysteine (NAC) is the precursor of l-cysteine and therefore of reduced glutathione and has been widely used as an antioxidant.

### Intracellular oxidative stress

**Figure 3:**
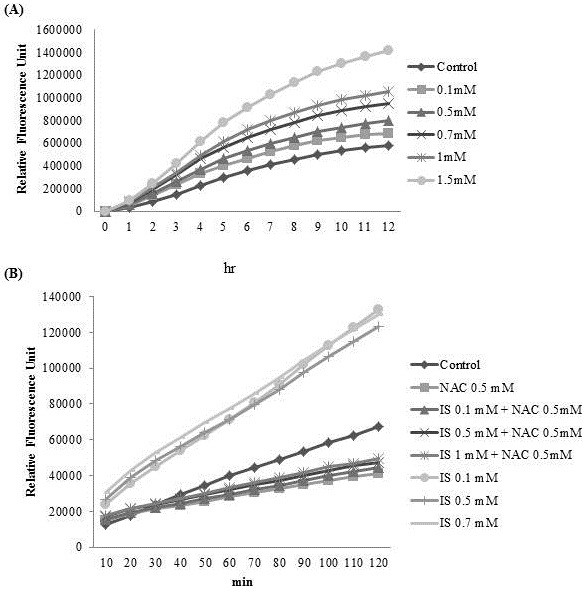
**Free radical production induced by the addition of IS.** MC3T3-E1 cells were seeded in 96-well plates, IS was added, and free radical production was measured after the indicated time. **(A)** Free radical production increased with time in a dose-dependent manner. **(B)** IS-induced free radical production was suppressed by 0.5 mM *N*-acetylcysteine (NAC). The data represent the mean from six replicates ns in each group.

As shown in Figure 3, IS increased cellular oxidative stress in a concentration-dependent manner. Addition of antioxidants or the OAT inhibitor suppressed free radical production (Figure 2C, Figure 3).

### Inhibition of osteoblast differentiation by IS

**Figure 4:**
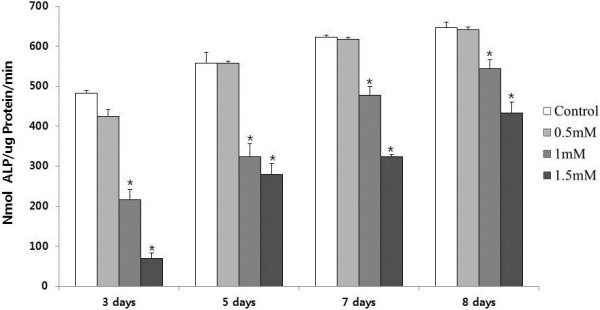
**Effect of IS addition on ALP activity in MC3T3-E1 cells.** ALP activity was suppressed by IS at concentrations greater than 1 mM. ALP activity is expressed as nanomoles of *p*-nitrophenol released per milligram of protein. ALP, alkaline phosphatase *P < 0.05 vs. control cells at each time point. The data represent the mean ± SEM (n = 6 for each group).

**Figure 5:**
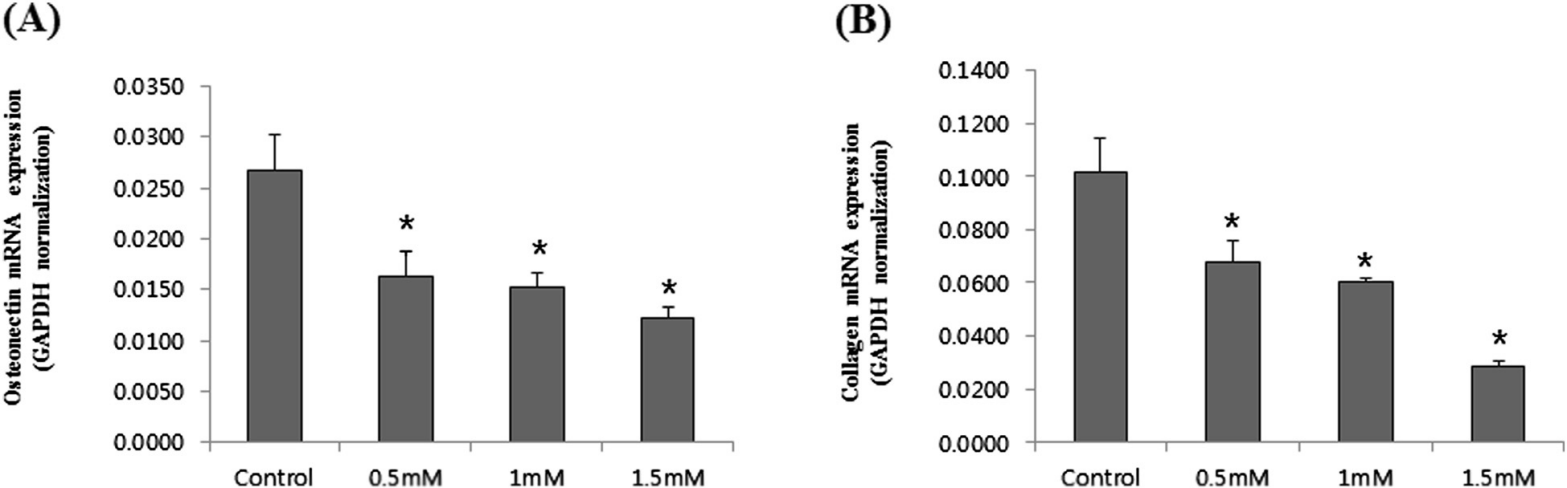
**Changes in collagen 1 and osteonectin gene expression in MC3T3-E1 cells after IS addition. (A)** Expression of the osteonectin gene upon treatment with various concentrations of IS at 5 d. **(B)** Expression of the collagen 1 gene upon treatment with various concentrations of IS at 5 d. *P < 0.05 vs. control cells. The data represent the mean ± SEM (n = 4 for each group).

To determine the differentiation of the pre-osteoblast cell line, ALP activity was measured in osteogenic induction medium with or without IS. As shown in Figure 4, ALP activity was suppressed above 1 mM IS. Collagen 1 and osteonectin were produced only in differentiated osteoblasts. To determine whether the osteoblasts had differentiated, the expression of collagen 1 and osteonectin mRNA was analyzed using real-time PCR. At 5 d, the production of collagen 1 and osteonectin mRNA was significantly inhibited by the addition of IS, as shown in Figure 5.

### Apoptosis induction by IS

**Figure 6:**
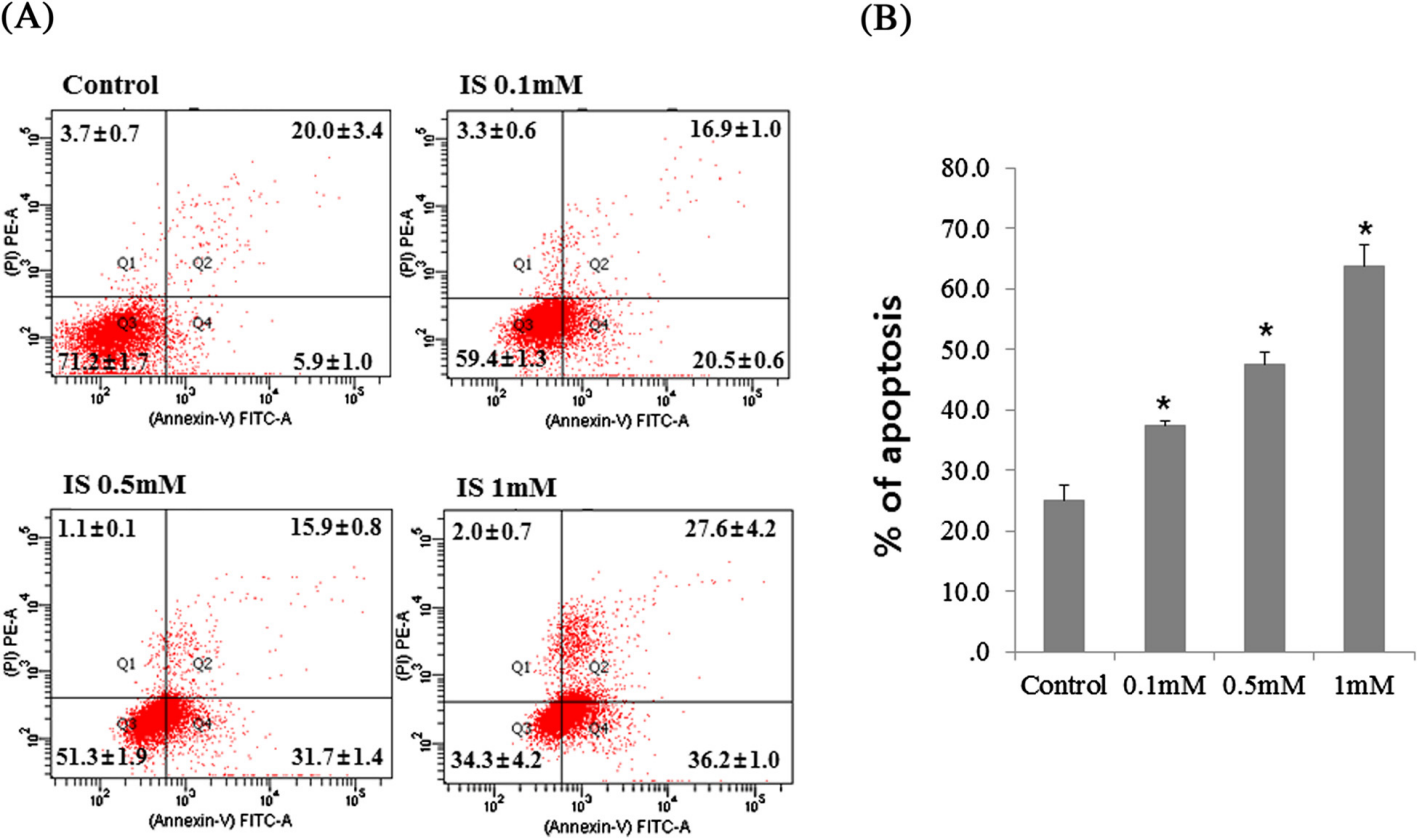
**Effect of IS on MC3-T3 cell apoptosis, determined by FACS analysis. (A)** Cells were incubated with various concentrations of IS for 24 h, after which they were harvested, the DNA was stained with propidium iodide, and the cells were analyzed using FACS. **(B)** IS increased the proportion of apoptotic cells in MC3-T3 cells. *P < 0.05 vs. control cells. The data represent the mean ± SEM (n = 3 for each group).

**Figure 7:**
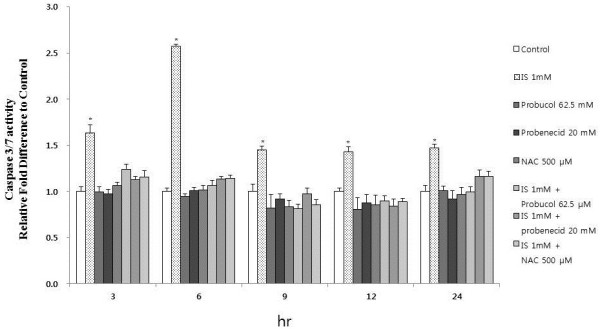
**Effect of IS on caspase-3/7 activity.** Caspase-3/7 activity was analyzed using a Promega Caspase Glo 3/7 kit (described in the Materials and Methods) Caspase-3/7 activity peaked at 6 h at 1 mM IS. IS increased caspase- 3/7 activity, but probenecid (20 mM), an OAT inhibitor, attenuated IS- induced caspase-3/7 activity, which is similar to an antioxidant effect. The data represent the mean ± SEM (n = 6 for each group). *P < 0.05 vs. control cells at each time point.

**Figure 8:**
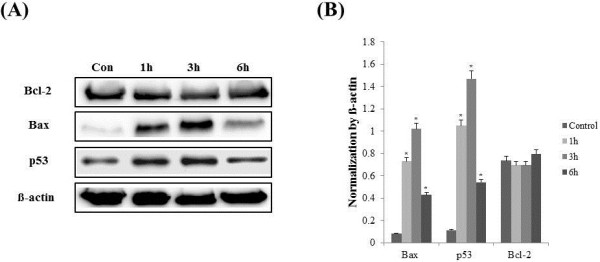
**Effect of IS on the protein expression of Bcl-2, Bax, and p53 in MC3-T3 cells. (A)** Representative immunoblots with anti-Bcl-2, anti-Bax, anti-p53, and anti-β-actin at 1 mM IS. **(B)** Densitometric analysis of Bcl-2, Bax, and p53, normalized by the data for β-actin. The data represent the mean ± SEM (n = 3 for each group).

To determine whether IS induces apoptosis, the cells were incubated with different concentrations of IS for 12 h, stained, and subjected to Fluorescence-activated cell sorting (FACS) analysis to measure apoptosis and necrosis. IS increased the proportion of apoptotic cells, particularly in osteoblasts (Figure 6). To elucidate the role of caspases in osteoblasts, we first examined the activity of the executioner caspase-3/7 in response to IS in osteoblasts. The activity of caspases was determined using fluorometric peptide substrates specific to caspase. As shown in Figure 7, the activity of caspase-3/7 peaked at 6 h of incubation with IS (1.0 mM). Caspase-dependent apoptosis thus appears to be involved in IS-induced osteoblast toxicity. To determine which apoptosis-related factors may be acting upstream of caspase activation, the expression of p53, Bcl2, and Bax was measured after the addition of IS (1.0 mM). IS increased the expression of Bax and p53, which play a role in apoptosis. However, Bcl-2 was not influenced by IS (1.0 mM) at 1, 3, and 6 h (Figure 8).

## Discussion

In the present study, we studied whether the uremic toxin IS directly suppressed osteoblast differentiation and induced osteoblast apoptosis via caspase activity. Bone toxicity is mediated by IS-induced free radical production, which evokes apoptosis. Our data emphasize the fact that several uremic toxins could affect osteoblast differentiation and function. Recently, several reports have shown that IS may be a bone toxin [8-10]. Iwasaki et al. [8] showed that oral administration of the indole-absorbing Kremezin prevents the progression of renal failure and improves bone formation in a rat model of chronic kidney disease (CKD) with low bone turnover. This group also demonstrated that the IS taken up by osteoblasts via the OAT-3 present in these cells augments oxidative stress to impair osteoblast function and downregulate PTH receptor expression [9]. These findings suggested that IS may be a bone toxin that is taken up via OAT-3 in osteoblasts and inhibits bone turnover by free radicals produced because of IS. Our data support the previous studies and suggest that IS itself could induce apoptosis via the production of free radicals.

Our data showed that IS-induced apoptosis is mediated by caspases. It is well established that p53 positively regulates Bax, but negatively regulates Bcl-2 expression [13]. IS-elicited changes in the Bax protein level are likely to be the result of an increase in p53 [14]. The Bcl-2 family plays a prominent antiapoptotic role by acting upstream of caspase activation. Our data showed that the expression of Bcl-2 is not influenced at 1, 3, or 6 h. The proapoptotic factors Bax and p53 may play a predominant role in IS-induced osteoblast apoptosis, which is activated via the caspase pathway.

Mozar et al. showed that IS inhibits osteoclast differentiation and bone-resorbing activity [10]. Goto et al. [15] reported that IS levels correlated negatively with 2 serum markers of bone formation (alkaline phosphatase and bone-specific alkaline phosphatase) independently of intact PTH, but not with a serum marker of bone resorption (TRAP 5b). Although serum markers may not be ideal for testing bone turnover, on the basis of their results, they suggested that IS may promote low-turnover bone disease outcomes (such as adynamic bone disease) observed in CKD patients.

With CKD progression, the ability of the kidneys to maintain systemic mineral homeostasis gradually decreases, resulting in the various abnormalities of bone and vascular physiology observed in CKD-MBD (CKD associated with mineral and bone disorders) [16]. In high-turnover bone disease, e.g., secondary hyperparathyroidism in CKD patients, PTH can be controlled by many drugs, including phosphate binders, vitamin D, and cinacalcet. However, no treatment specific to low-turnover bone disease is available. Elimination of uremic toxins could be an option for controlling low-turnover bone disease in CKD patients.

Our study suggested that IS-induced apoptosis might be mediated by free radical production. These findings are supported by many previous reports [15,16]. Several studies have shown that IS induces NADPH oxidase mRNA expression and increases its activity in various types of cells [17-19]. Namikoshi et al. reported that decreasing the serum concentration of IS by administrating the oral adsorbent AST-120 reduces the expression of the NADPH oxidase component and alleviates oxidative stress in the aorta [20]. In our study, we did not determine NADPH mRNA expression or its activity, but it appears that increasing free radical production by adding IS may occur through an activation pathway of NADPH oxidase.

A list of uremic toxins has been provided by EUTox. The normal concentration of IS is 0.53 mg/L (2 μM). The mean concentration of IS in uremic patients is 23.1 mg/L (~100 μM), and the maximum concentration found in uremic patients was 44.5 mg/L [21].

## Conclusions

Our data confirm that IS acts as a bone toxin by inhibiting osteoblast differentiation and inducing apoptosis via the caspase pathway. Further studies are required to elucidate whether elimination of IS could improve osteoblast differentiation in chronic renal failure.

## Abbreviations

ALP: Alkaline phosphatase; DCF-DA: 2′-7′-dichlorofluorescein diacetate; CKD: Chronic kidney disease; CKD-MBD: CKD associated with mineral and bone disorders; IS: Indoxyl sulfate; MTT: 3-[4,5-dimethylthiazol-2-yl]-2,5-diphenyl-tetrazolium bromide; OAT: Organic anion transport; PNP: p-nitrophenol; PTH: Parathyroid hormone; RIPA: Radio-immunoprecipitation assay; ROS: Reactive oxygen species.

## Competing interests

All the authors declare that they have no competing interests.

## Authors’ contributions

KYH carried out the molecular studies and PCR array. KKA carried out the molecular studies, FACS scan. GHW conceived of the study, and participated in its design and coordination. SHY participated in the design of the study and performed the statistical analysis. SHY participated in the design of the study and performed the statistical analysis. All authors read and approved the final manuscript.

## Pre-publication history

The pre-publication history for this paper can be accessed here:

http://www.biomedcentral.com/2050-6511/14/60/prepub
